# Child psychiatry: a model for specific goals for in-patient treatment linked to resources and limitations in out-patient treatment

**DOI:** 10.1192/bjb.2020.29

**Published:** 2020-12

**Authors:** Simon R. Wilkinson

**Affiliations:** National Centre for Child Psychiatry, Oslo, Norway

**Keywords:** In-patient treatment, psychiatric nursing, out-patient treatment, personality disorders, family therapy

## Abstract

I present a rationale for two different types of in-patient child psychiatric unit: 24/7 intensive units and 24/5 child and family units. Intensive units address safety requirements. The developing personality of young people is at the centre of in-patient approaches on the child and family units. This requires attachment-informed practice. Families must always be involved and placement of units must facilitate their participation. The primary skill characterising these units is use of the milieu for therapy and combining this with family therapy. In other words, nurses and allied professionals need to be the dominant force in unit development, under the reflective guidance of consultants and clinical psychologists.

Sitting beside a nurse from a county in California at an international conference I realised for the first time that areas in the USA provide third-world levels of child psychiatric treatment. She was the only clinical worker for children and adolescents with psychiatric problems for a county with a total population close to 100 000. She arranged for patients to fill out forms, which were then sent to an adult psychiatrist, who prescribed medication over the phone. My concern is that general UK provision has for a long time been critically vulnerable because of the extremely low staffing levels and lack of obligation to provide a comprehensive service within each health trust. I do not believe that the UK model of service provision is conducive to making a leap forward with services tied to a tiers model. Yet there is no evidence of what ingredients might promote a healthier service. Even though we have evidence that in-patient treatment can give benefits,^1^ we have no conceptualisation of how the complex interconnected processes on an in-patient ward might be working. Instead I will present here an analysis of how need can be formulated to suggest an alternative service model which can identify the elements that need addressing. Although I have been working within the well-endowed Norwegian health service, and there is no chance of the UK approaching the same level of provision in the near or distant future (we have over ten times the resources compared with the UK and other wealthy countries^2^), analysis of what is required is no different. Having worked outside the taken-for-granted world of UK child psychiatry, a different perspective can be generated which maybe leads to new possibilities.

## Safety first – 7 days a week (24/7)

The top priority has to be given to ensuring safety, when, by virtue of the young person's mental state, the patient or their VIPs (I will use ‘very important person’ to cover the adults responsible for ensuring the patient's care – biological parents, foster parents, etc.) have been in danger. This has to be available 24/7 and it requires a mix of out-patient provision and emergency out-of-hours service in liaison with a unit available to admit patients 24/7. The issue of whether it is desirable to provide more than 09.00–17.00 h out-patient provision to enable both VIPs and patients easier access to specialist services needs to be debated. Or could emergency services be staffed with child and adolescent psychiatric expertise? (In Oslo there is a psychiatric emergency department which is staffed until 23.00 h, covering the time after out-patient departments cease their obligation to evaluate emergencies at their closing time. Some privileged populations in the UK have access to services with such out-of-hours specialist cover.) Why is it not expected that all emergency departments should be catering for the second highest cause of mortality among our adolescents after accidents?

Rather than going into the pitfalls of evaluating potentially suicidal behaviour, as only one potential reason for acute referral, I will only remind us here that those with greater experience refer fewer patients for admission because of potential suicide. This underlines why highest-quality expertise is needed in the front line.

It is not the Axis I diagnosis that determines the need for admission, but the necessity of an alternative ‘containment’ of the patient to that available at home. Two factors operate here: the state of the child and the processes at home. The ICD-10 system's Axis V diagnoses of the psychosocial context were included as of prognostic importance and needed to be addressed in treatment plans. Context matters as much as the young person's ‘state’ – and it is entwined in the same complex causal system, such that states evolve to ‘traits’ depending on the maintaining influence of context processes. No admission should occur without the possibility of integrating approaches to the child's state, as well as addressing precipitating and maintaining factors in the context of the episode. The younger the patient, the more important the context factors in treatment. Here, planning for young people differs from that for adults. Out-of-region ‘refuse recirculation’ is detrimental to patient recovery and welfare and can be expected to be associated with need for more frequent readmissions. Every such unit needs to be able to work with family, school and local resources – and that means fathers as well as mothers.

The critical elements concern ensuring highest expertise during out-patient evaluations, with ideally similar levels of expertise available ‘out of hours’, and an in-patient resource that is equipped to address the patient's state as well as the context factors. The level of staffing needs to be high – and, importantly, consist of a stable group of nurse therapists (use of locums is countertherapeutic to the teamwork required). A higher level of staffing and greater stability of staff leads to reduced needs for restraint or emergency medication.^a^ Staffing needs to include other therapists working in the patient ‘milieu’^b^ who are expert in childcare issues, such as residential social workers. They need to be used to working with complicated family dynamics, where abuse of power – both verbal and physical – may well be a problem. Safety is primarily ensured by these staff, to a greater degree than by doctors and clinical psychologists, who provide the back-up understanding of the treatment needs and reflection on the treatment dynamic as it evolves. The high staffing levels enable the intensive approach required, and this suggests that the proposed 24/7 acute admission units should be called intensive units, as in UK adult psychiatry. Because transfer of patients between units and therapists creates lacunae of security, such intensive units should be able to keep treating their patients who require longer than an acute admission. This most vulnerable group of patients needs help to set up their transfer back to the context from which they were admitted, so that a return to their out-patient department will succeed without the patient isolating themselves in connection with the transition. An adequate model for practice needs to include an ambulant service from the intensive unit to anchor the patients in meaningful activities at home – school, clubs, sports, etc. – so that any tendencies to isolation can be mitigated.

## Working with personality – 5 days a week (24/5)

There is another group of patients who can also benefit from admission, albeit not the intensive kind with a focus on security. Often these have been referred for diagnostic evaluation and treatment. But in principle there is little to suggest that an admission should be used for diagnosis, given the skills that need to be available on an out-patient basis (in my practice the costs of 1 year's admission was the equivalent of 4–5 full-time out-patient clinicians). What cannot be done there? Out-patient clinicians cannot easily evaluate problems arising at bedtime and during the night! Usually such referrals for diagnosis are a ‘cop out’. Any referral precipitated by lack of progress in out-patients requires that the covering letter identifies the barriers preventing progress – and how these might be more successfully addressed during an admission. In my experience this does not happen. Additionally, on admission, objective criteria should be decided as to how far treatment needs to have come before transfer back to out-patient provision can occur successfully.

Do we know what the barriers are likely to be? They are not the Axis I diagnoses, as all of these can potentially be treated outside institutional services. I have my experience to go on from Norway, and it may be very different in the UK. My guess is that the barriers are universal, but seldom acknowledged. The elephant in the room, the dimension not talked about in child psychiatry, is personality, both that developing in the patient and in the VIPs. Progress in treatment depends on working with both the patient and the VIP and establishing an alliance with them that facilitates their ability to take chances in doing things differently. But this is easier for some than others. The more insecure a VIP or patient the greater their need to do things to feel in control of the situation. These individuals can be impulsively dramatic. And they take fewer chances to do things differently – for the VIP to read the child's signals differently, to understand their vulnerabilities in new ways, or for the child to respond to their fears as much as to fight their way out of a corner, to take chances to tell others what they are going through. In such control-focused meetings things continue to happen impulsively with little reflection, according to well-worn strategies. As Peter Cook replied to Dudley Moore when asked what he had learnt from his mistakes: ‘I could repeat them exactly’. This tendency to impulsiveness associated with lack of change can easily suggest to the therapist that they are missing something and that a diagnostic admission is required. Instead I suggest there is a need for an admission to a totally different sort of unit than the intensive unit. There will be no sole focus on the Axis I diagnosis, Axes II–V being at least as significant for the admission – and assuming that Axis VI scores (Children's Global Assessment Scale, CGAS) are at least under 40. Admitting patients with primarily personality problems to an intensive unit is expected to be counterproductive.

A child and family unit can have a lower staffing level. It is not apparent to me that it should be a 24/7 unit, as there are advantages of the patients being at home at weekends. Therefore a 24/5 unit would be better. It needs to be able to address the impulsive responses to which the patients and their VIPs are disposed, fuelled from their implicit memories. The milieu staff would interact with patients in the ‘here and now’, allowing response patterns characteristic of the dispositional representations^3^ of the patients’ personalities to be revealed, and would set up situations to create optimal learning opportunities. The staff would be grounded in understanding of personality, a field with which child psychiatry is only slowly coming to grips. My suggestion is that adapting Clarkin's^4^ conceptualisation of personality and informing it with knowledge about child development will give us personality as reflecting factors present at birth or by the end of the first years of life (Clarkin used the short-hand ‘temperament’, as if it were an obviously genetic factor) in interaction with attachment strategies, and influenced by experiences of trauma and loss. This would have given us the personality dimensions suggested, but not adopted, for DSM-5.

The advantage of such a unit operating 24/5 is that the focus is continually on patterns that recur in the home environment. Has the treatment week led to different patterns at the weekend? During the week it is necessary to work with the patient, but also with parental responsiveness and the parent's ability to identify precipitating and maintaining factors in their child's disturbance. Each weekend is not a break from treatment, but a time for renewal and update of the treatment contract, refreshing and refining the focus for all concerned. With clarity preceding admission in what needs to have changed before out-patient treatment can resume, the approaching discharge timing will be clear to all involved ‘en route’. If issues of security arise during the weekends then a temporary move to an intensive unit may be required, but the priority is a thorough analysis of the sequences that precipitated the situation by the staff from the 24/5 unit.

These units will often find they are working in the no man's land between child-protection services and medicine. Child psychiatric services will always need to have close liaison with social services and make use of consultation from child-protection services – and provide corresponding consultation to child-protection services.

## A plea

From my position across the water it is indefensible to be bussing young patients out of region. Every patient should be getting eventual need for admission met within easy travel distance of home so that family approaches can be integrated with the in-patient treatment. If a unit is designated as an intensive unit it should not be allowed to close its doors to local acute admissions. It should be under an obligation to provide the 24/7 service required even if it means temporary beds. Any other arrangement rewards keeping patients longer than required to enable the unit to avoid the crushing work of new admissions; and frees the out-patients from pressure to ensure a rejuvenated service for the returning patient. Tier 4 expertise should be available primarily on an out-patient basis. An admission is not to a hierarchically superior service, but to one of two possible treatment units providing distinctly different provision, where the role of the milieu staff has primacy in the treatment strategy.

## Footnotes

a. The Norwegian adaptation of the Quality Network for Inpatient CAMHS (QNIC), known as KvIP (www.akuttnettverket.no/kvip-barn-og-unge), has found that an emergency sedation regime has not proved necessary, and it was recently removed from the list of standards. This is attributed to the higher levels of stable qualified staffing.

b. In Norway the staff are collectively known as milieu therapists, and include both nurses, childcare workers and other professionals, besides the non-milieu psychologists and child psychiatrists.

## About the author

**Simon Wilkinson**, MD, FRCPsych, has run the adolescent in-patient units at the National Centre for Child Psychiatry, Oslo, Norway. He has retired from clinical practice although is still employed by Oslo University Hospital and Akershus University Hospital to provide psychotherapy supervision for junior doctors in training.
